# A highly sensitive octopus-like azobenzene fluorescent probe for determination of abamectin B_1_ in apples

**DOI:** 10.1038/s41598-021-84221-w

**Published:** 2021-02-25

**Authors:** Zhenlong Guo, YiFei Su, Kexin Li, MengYi Tang, Qiang Li, Shandong Xu

**Affiliations:** 1 Department of Chemistry, College of Science, Beijing Forestry University, Beijing, 100083 China; 2 College of Biological Sciences and Biotechnology, Beijing Forestry University, Beijing, 100083 China; 3 Beijing Key Laboratory of Forest Food Processing and Safety, Beijing, 100083 China

**Keywords:** Risk factors, Analytical chemistry

## Abstract

The development of detecting residual level of abamectin B_1_ in apples is of great importance to public health. Herein, we synthesized a octopus-like azobenzene fluorescent probe 1,3,5-tris (5′-[(*E*)-(*p*-phenoxyazo) diazenyl)] benzene-1,3-dicarboxylic acid) benzene (TPB) for preliminary detection of abamectin B_1_ in apples. The TPB molecule has been characterized by ultraviolet–visible absorption spectrometry, ^1^H-nuclear magnetic resonance, fourier-transform infrared (FT-IR), electrospray ionization mass spectroscopy (ESI-MS) and fluorescent spectra. A proper determination condition was optimized, with limit of detection and limit of quantification of 1.3 µg L^−1^ and 4.4 μg L^−1^, respectively. The mechanism of this probe to identify abamectin B_1_ was illustrated in terms of undergoing aromatic nucleophilic substitution, by comparing fluorescence changes, FT-IR and ESI-MS. Furthermore, a facile quantitative detection of the residual abamectin B_1_ in apples was achieved. Good reproducibility was present based on relative standard deviation of 2.2%. Six carboxyl recognition sites, three azo groups and unique fluorescence signal towards abamectin B_1_ of this fluorescent probe demonstrated reasonable sensitivity, specificity and selectivity. The results indicate that the octopus-like azobenzene fluorescent probe can be expected to be reliable for evaluating abamectin B_1_ in agricultural foods.

## Introduction

Avermectins, being one type of macrolide antibiotics, have been widely used as bactericide, insecticide and miticide for plants or animals, which have excellent characteristics of disturbing the target's neurophysiological activities and can easily be decomposed by soil microorganisms^1^. Abamectin B_1_, is the only avermectin that has been widely approved for plants and animals because of its efficient antiparasitic activity^2,3^. However, the spread use of avermectins tends to result in consecutive accumulation in food-producing animals and plants^4,5^. To detect avermectins (Abamectin B_1_), various analytical approaches were employed, involving high-performance liquid chromatography-ultraviolet detection (HPLC–UV)^4^, liquid chromatography-tandem mass spectrometry (LC–MS)^6^, high-performance liquid chromatography-fluorescent detector (HPLC-FLD)^7^, enzyme-linked immunosorbent assay (ELISA)^8^ and liquid chromatography-tandem mass spectrometry/mass spectrometry (HPLC–MS/MS)^9^. Whereas, in most case, these approaches either needed a time-consuming process, high-cost accurate instrument, and high professional operators, which makes them difficult to apply in general laboratories^10^. Herein, considering the potential harm of abamectin B_1_ for public health, a convenient and accurate analysis method for residual abamectin B_1_ is necessary^10,11^.

Fluorescent probe has gained great attention in recent years owing to high sensitivity, high selectivity, fast response, low cost, and direct detection^12^. Especially, azobenzene fluorescent probe exhibits outstanding fluorescent quantum yield, light stability^13^, and chemical and thermal stability^14^. Fluorescent probe has been applied to analyze organophosphorus pesticides^15^, organochlorine pesticides^16^ and carbamate pesticides^17^. However, there are few reports on fluorescent probe for monitoring avermectin residual in literature due to the weaker fluorescence signal of a single chromophore^18^ and the difficulty in recognizing their complicated chemical structures containing ketones, aldehydes and hydroxyl groups^19^. Therefore, a fluorescent molecule, with rationally designed structures, containing multiple chromophores and recognition groups, is an ideal probe to monitor avermectin B_1_.

**Figure 1:**
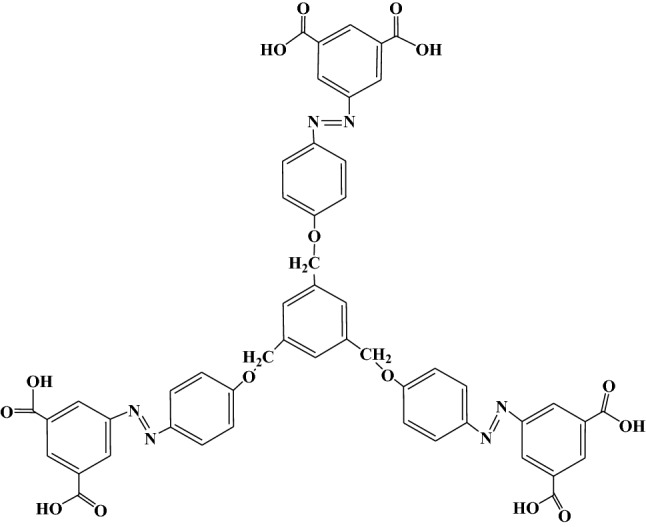
Structure of single molecular TPB.

Herein, this study reported the synthesis of octopus-like 1,3,5-tris (5′-[(*E*)-(*p*-phenoxyazo) diazenyl)] benzene-1,3-dicarboxylic acid) benzene (TPB), with six carboxyl groups and three azo chromophores (Fig. 1). Its application as a fluorescent probe was proved to be feasible by evaluating its fluorescence properties. A sensitive and specific approach was further established for qualitative and quantitative assay of abamectin B_1_. The applicability of approach was evaluated in apple samples, based on a visible fluorescence signal for this probe towards avermectin B_1_ at 420 nm.

## Results and discussion

### Fluorescence properties of TPB

**Figure 2:**
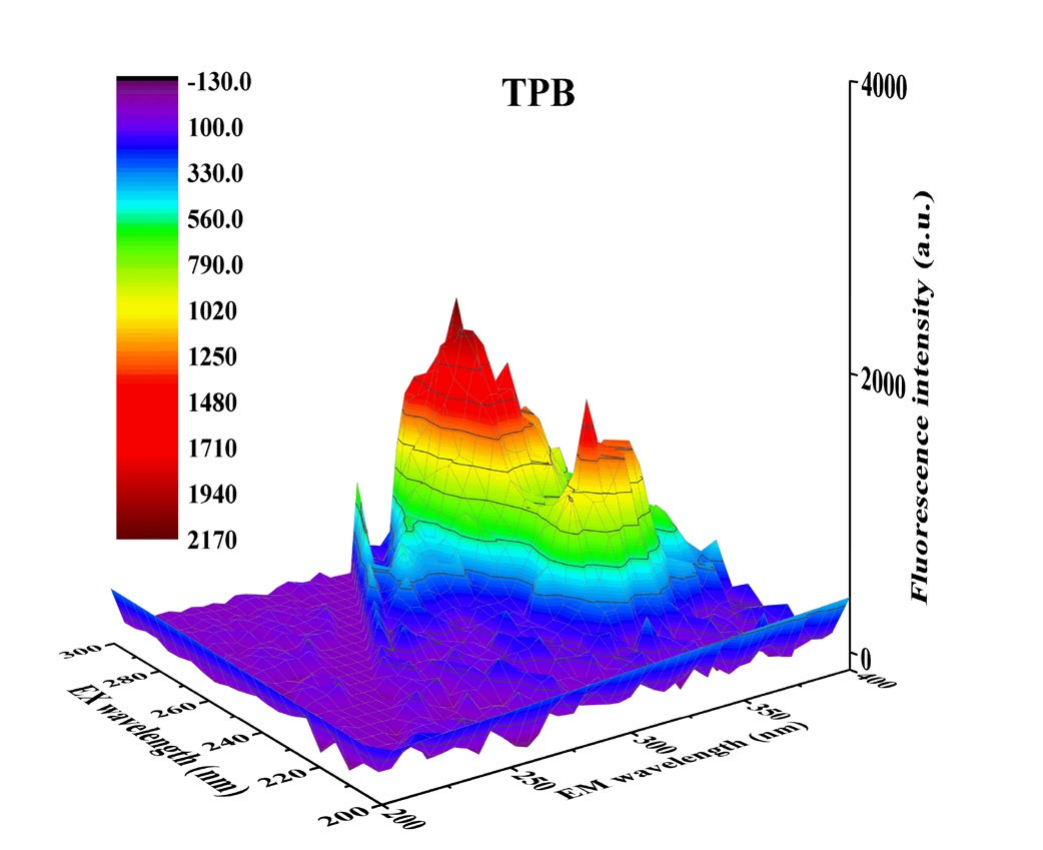
Fluorescence 3D contour spectra of TPB (0.006 mmol L^−1^). Excitation wavelength: 200–600 nm, Emission wavelength: 200–600 nm.

**Figure 3:**
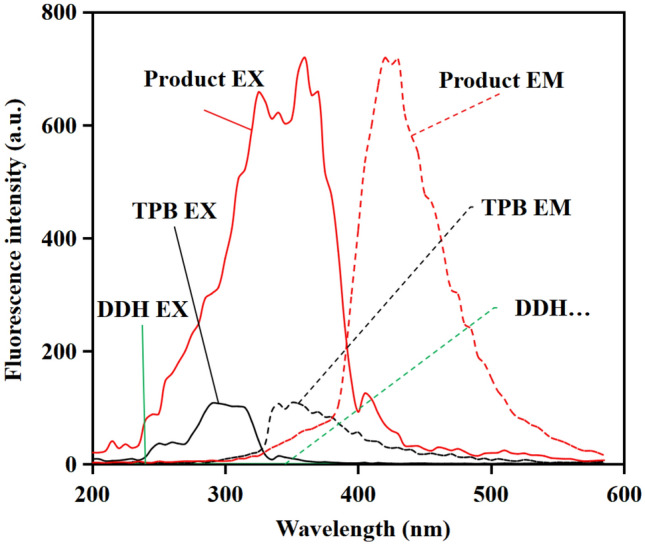
Comparison of fluorescent excitation (EX) and emission (EM) spectra of DDH (0.006 mmol L^−1^), TPB (0.006 mmol L^−1^) and the product (0.01 mg L^−1^). Excitation wavelength: 200–600 nm, Emission wavelength: 200–600 nm.

The fluorescence property of TPB was investigated, with its precursor 5-(4-hydroxyphenylazo)-isophthalic acid dimethyl ester (DDH) (chemical structure was shown in Figure S1) for comparison, which only has one azo chromophore. When TPB and DDH were irradiated by ultraviolet light, TPB displayed a maximum emission at 350 nm and excitation at 290 nm (Fig. 2), while no fluorescence signal was visible for DDH (Fig. 3). The difference of fluorescence property was attributed to the more released energy of TPB than that of DDH when the excited state electrons returned from the excited singlet state (S) to the spin singlet electron (S_0_), which was based on the superposition of fluorescence effect of three azo chromophores. Furthermore, improved fluorescence property of TPB was ascribed to enhance resistance to internal rotation of molecule resulting from the introduction of 1,3,5-tris (bromomethyl) benzene and the expansion of space system effect. Moreover, the Stokes shift of TPB was calculated to be 60 nm. The Stokes shift, was considered to effectively decrease detection errors, resulting from the interference from auto-fluorescent of samples^24^ and the spectral overlap between the fluorescent and excitation light^25^. Therefore, TPB was expected to be suitable for employ as a fluorescent probe.

We further investigated the applicability of TPB to abamectin B_1_. Upon the addition of abamectin B_1_, the emission band at 350 nm and the excitation band at 290 nm of the probe shifted forward to 420 nm and 360 nm respectively (Fig. 3). Generally, different fluorescence molecules have different excitation and emission spectra, which can determine the specificity and selectivity of analysis by using fluorescent probe. The Stokes shift of 60 nm was calculated and reflected rational anti-interference ability. This result confirmed that abamectin B_1_ could be identified by probe TPB under the certain conditions, which indeed underpinned qualitative analysis of abamectin B_1_.

**Figure 4:**
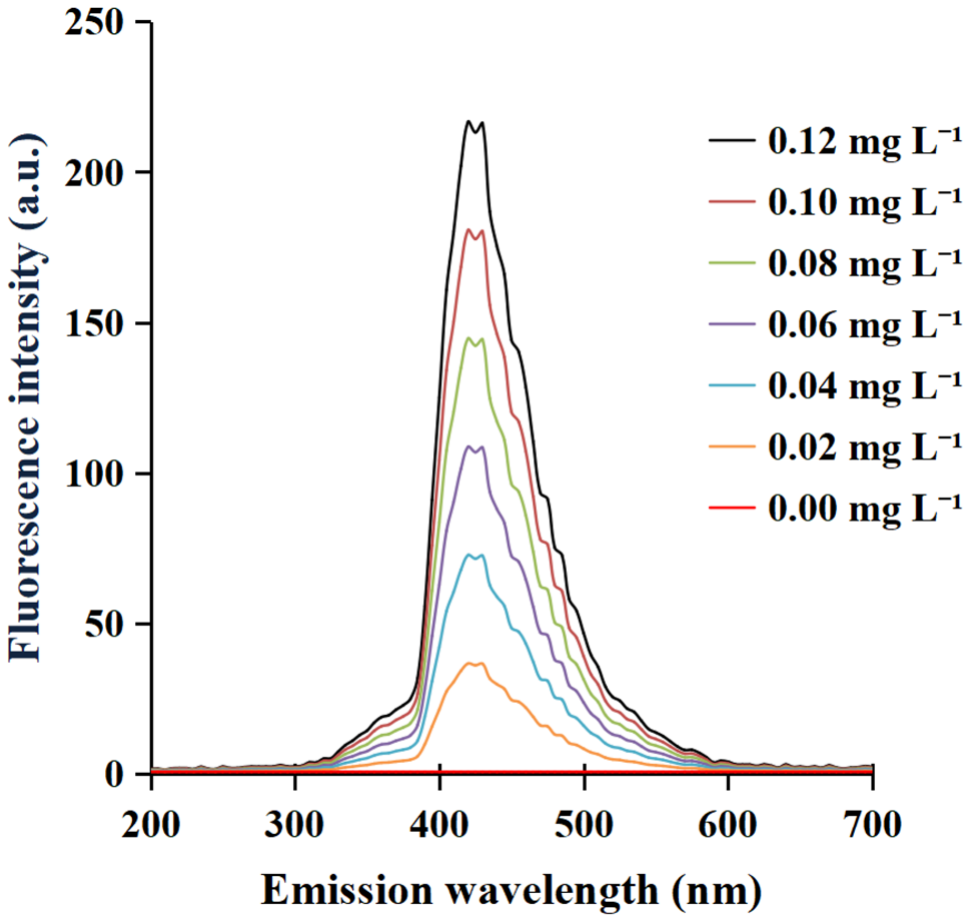
Comparison of fluorescence spectra of the product with 0.00–0.12 mg L^−1^ abamectin B_1_. Excitation wavelength: 360 nm, Emission wavelength: 200–700 nm.

Furthermore, the fluorescence intensities of the reacted product at 420 nm were examined at different concentrations of abamectin B_1_. Figure 4 presented that the fluorescence intensity of the reacted product at 420 nm were enhanced gradually with the increasing concentration of abamectin B_1_. The which was considered as the basis of quantitative analysis. Therefore, TPB could be used as a fluorescence probe for assessing the level of abamectin B_1_.

In the absence of abamectin B_1_, probe TPB had no or faint fluorescence, otherwise it emitted the visible fluorescence (Fig. 4), which confirmed the probe TPB was the typical fluorescent molecule based on the effect of Photoinduced Electron Transfer (PET). Further analysis for TPB’ structure showed that its carboxyl groups, azo groups and peripheral phenyls might be considered as receptors, fluorophores and spaces respectively. Based on frontier orbital theory, in the existence of abamectin B_1_ and its relevant substances with the same recognized group, probe TPB should engender different LUMO and HUMO, which leaded to unequal excitation and emission spectra. Therefore, probe TPB has a good sensing selectivity.

### The mechanism of identifying abamectin B_1_ by TPB

To investigate the mechanism of identifying abamectin B_1_ by TPB, fluorescence spectroscopy, FT-IR and ESI-MS were employed to compare the difference of the TPB before and after adding abamectin B_1_. After adding abamectin B_1_, FT-IR spectra showed additional peaks at 1704 cm^−1^ and at 1149 cm^−1^ (Figure S2), corresponding to the ester group. The appearance verified the recognition of probe TPB to abamectin B_1_ achieved by esterification. In addition, a clear red shift of wavelengths in fluorescence spectroscopy was noticed (Fig. 3), which was assigned to the changed conjugate systems of electron-donating and rearranged internal charges prompted by enhanced ability of TPB to capture abamectin B_1_. Moreover, ESI-MS spectra yielded a peak at *m*/*z* = 2717.1910 (Figure S6), which corresponded to a new product produced between probe TPB and abamectin B_1_, thus underlying the mechanism of detection.

**Figure 5:**
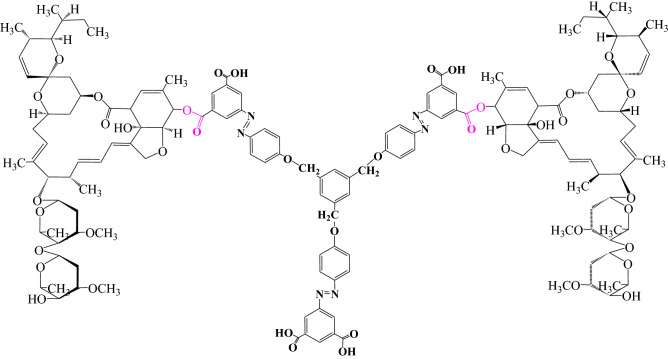
Recognized mode of the reacted product.

For abamectin B_1_, the hydroxyl groups at the C_5_ and C_7_ are allylic, which should have the high reactive. However, the allylic hydroxyl group at the C_7_ A is too easily forming hydrogen bonds with adjacent ester groups and too sterically hindered to be reactive^26^. Hydroxyl group at the C_4_ is a general, and its activity is weaker than that of C_5_ and C_7_. Thus, only hydroxyl group at the C_5_ has the potential to be used for esterification theoretically. In addition, due to the effect of steric hindrance on TPB, its combination with abamectin B_1_ can only take place in the counterpoint. However, on the same phenyl of TPB with two carboxyl groups, in the existence of one abamectin B_1_, the adjacent carboxyl groups will be passivated and incapable to add another abamectin B_1_. Therefore, a recognition mode was tentatively proposed, i.e., the two carboxyl groups of the octopus-like azobenzene fluorescent probe TPB were used as recognition sites to abamectin B_1_ with a molar ratio of 1:2 (Fig. 5), which was further confirmed by ESI-MS spectra with a peak at *m/z* = 2717.1910 (Figure S6).

### Establishment and evaluation of the method

To optimize the determination conditions, effects of different levels of the pH and amount of phosphate buffer were investigated. Figure S7 showed that the fluorescent intensity of the reacted product at 420 nm was initially increased at pH 5.0–6.0, followed by a decrease at pH 6.0–9.0, and eventually by the maximum at pH 6.0. The increase–decrease–maximum trend reflected the proper protonation of carboxyl groups of TPB facilitated the coordination process and enhanced the nucleophilicity of hydroxyl of TPB to abamectin B_1_. Figure S8 showed that the fluorescent intensity of the product at 420 nm was initially increased at 0.2–0.8 mL phosphate buffer (0.2 M, pH 6.0), followed by a stabilization at 0.8–1.2 mL phosphate buffer (0.2 M, pH 6.0). Therefore, the validation of using probe TPB was estimated at 0.8 mL phosphate buffer (0.2 M, pH 6.0), by optimizing the determination conditions, linear equation, correlation coefficients (R^2^), limit of detection (LOD), limit of quantification (LOQ) precision and linear range. Apparently, the fluorescent intensity of probe TPB mixing with various concentrations of abamectin B_1_ exhibited a good linear relationship at the linear range from 4.4 to 60 μg L^−1^, giving rise to a LOQ of 4.4 μg L^−1^ (Table S2). In addition, the LOD of the described method was calculated as 1.3 µg L^−1^ (Table S2).

**Figure 6:**
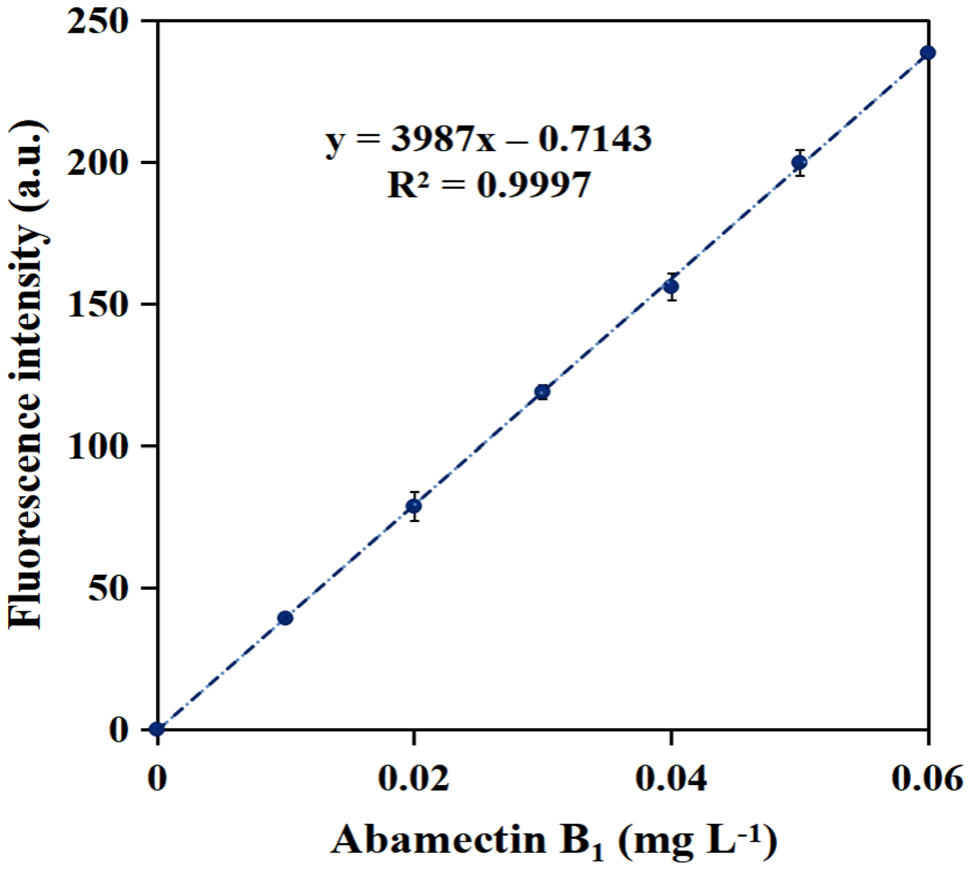
Plot of fluorescence intensity differences with 0.00–0.06 mg L^−1^ abamectin B_1_. Excitation wavelength: 360 nm, Emission wavelength: 420 nm.

The linear regression equation was thus determined to be Y = 3987X – 0.7143 (Fig. 6), where Y was the fluorescent intensity of probe TPB at 420 nm, and X represented the concentration of abamectin B_1_. Correlation coefficients (R^2^) was determined to be 0.9997, which manifested the satisfactory precision of the method. The enhancement clarified the suitability of this convenient and sensitive method for the determination of the abamectin B_1_ with tiny LOD, miniature LOQ, excellent precision and epic linear range.

Further comparison was conducted between our fluorescent probe method and those described previously. Table S3 showed that the recovery, linear range and LOQ of our fluorescent probe method were comparable to or superior to those of the methods reported previously.

A good reproducibility was represent by a relative standard deviation of 2.2% between eleven parallel experiments shown in Table S2. The final product had an unique fluorescence spectra at EX of 360 nm and EM of 420 nm, which could be considered to embody the specificity and selectivity of the method generally, as mentioned above. Sensitivity of the method was benefit from the six carboxyl recognition sites and three azo response sites of TPB.

### Preliminary detection of abamectin B_1_ in apple samples

Table 1 showed 2.0 μg L^−1^, 3.0 μg L^−1^ and 1.6 μg L^−1^ abamectin B_1_ in *Malus pumila* mill, Qinguan and Huangxiangjiao, respectively. To evaluate the accuracy of the proposed method, apple samples spiked with abamectin B_1_ at different levels of 4.4 μg L^−1^, 8.8 μg L^−1^ and 44 μg L^−1^ were determined. The recoveries of abamectin B_1_ spiked to apple samples were calculated in the range of 98.2–104.5% (Table 1), which indicated the suitability of this detection method in apples.

**Table 1:** Recovery values determination of abamectin B_1_ in apple samples of the approach.

Sample	Based level (μg L^−1^)	Added level (μg L^−1^)	Found average level (μg L^−1^)	Recovery (%)	RSD (%)
*Malus pumila* mill	2.0	4.4	6.4 (6.4, 6.4, 6.5)	100.0–102.3	0.9
	2.0	8.8	11.0 (11.1, 11.2, 10.7)	98.9–104.5	2.4
	2.0	44	45.5 (45.4, 45.2, 45.9)	98.2–99.8	0.8
Qinguan	3.0	4.4	7.5 (7.6, 7.6, 7.4)	100.0–104.5	1.5
	3.0	8.8	11.8 (11.8, 11.7, 11.9)	98.9–101.1	0.8
	3.0	44	47.2 (46.3, 48.2, 47.0)	98.4–102.7	2.0
Huangxiangjiao	1.6	4.4	6.1 (6.2, 6.0, 6.0)	100.0–104.5	1.9
	1.6	8.8	10.4 (10.4, 10.3, 10.6)	98.9–102.3	1.5
	1.6	44	46.3 (45.8, 46.2, 46.9)	100.5–103.0	1.2

## Conclusion

In summary, we had demonstrated the probe TPB synthesized for facile quantitative detection of the residual abamectin B_1_ in apples. The octopus-like TPB molecule was characterized using UV–Vis, ^1^H NMR, FT-IR, ESI-MS and fluorescent spectra. The determination conditions were tuned by varying different pH value and concentration, and a proper condition was achieved at pH 6.0 with LOD and LOQ of 1.3 µg L^−1^ and 4.4 μg L^−1^, respectively. The mechanism of the probe to identify abamectin B_1_ was tentatively proposed in a aromatic nucleophilic substitution through a combined mode of TPB and abamectin B_1_ with a molar ratio of 1:2. In particular, the facile quantitative detection of the residual abamectin B_1_ in apples was achieved. Our results showed that the novel approach of quantitative assay based on fluorescent probe is significative to evaluate the abamectin B_1_ in agricultural foods.

## Materials and methods

### Reagents and materials

Dimethyl 5-aminoisophthalate, 1,3,5-tris (bromomethyl) benzene, phenol, sodium acetate anhydrous, sodium nitrite, sodium hydroxide, *N*,*N*-dimethylformamide (DMF), ethanol anhydrous, methanol, magnesium sulfate anhydrous, potassium carbonate, sodium chloride, acetonitrile, tetrahydrofuran, hydrochloric acid, sulphuric acid, pH 4.0 phosphate buffer (0.2 M), pH 7.0 phosphate buffer (0.2 M), pH 8.0 phosphate buffer (0.2 M), pH 9.0 phosphate buffer (0.2 M), sodium dihydrogen phosphate anhydrous, sodium phosphate dibasic, potassium bromide, deuterated chloroform, octadecylsilane chemically bonded silica (C_18_) were purchased from Shanghai Macklin Biochemical Co., Ltd (Shanghai, China) and were of analytical grade. pH 6.0 phosphate buffer (0.2 M) was purchased from Beijing Lanyi chemical products Co., Ltd. (Beijing, China) and were of analytical grade. Quinine sulfate fluorescent standard substance (98.6%) and abamectin B_1_ were purchased from Aladdin Biochemical Technology Co., Ltd. (Shanghai, China). Demonized water (18 MΩ cm) was produced by using a water purification system (Water Purification System, Casccada 1, Pall, Beijing, China).

### General instrumentation

Fourier-transform infrared spectroscopy (FT-IR) was recorded on a Thermo 330 spectrometer at 500–4000 cm^−1^ wavelengths and a resolution of 3 cm^−1^ over 32 scans. The ultraviolet–visible absorption spectrometry (UV–Vis) was measured with a TECHCOMP UV2600 Spectrometer (TECHCOMP, Shanghai, China) at 200–800 cm^−1^ wavelengths. ^1^H-nuclear magnetic resonance (NMR) spectra was recorded on a JNM-ECA 600 MHz-NMR using tetramethylsilane (TMS) (JEOL, Japan). Electrospray ionization mass spectroscopy (ESI-MS) data of TPB and the product were obtained by using a Bruker New ultrafleXtreme MALDI-TOF mass spectrometer at NL: 7,990,000, RT: 4.58–4.66, AV: 34, T: FTMS-*p* ESI and NL: 41,300, RT: 2.13–2.22, AV: 36, T: FTMS-*p* ESI. Fluorescent spectra was performed using a Hitachi F-7000 spectrometer at Sampling Interval: 5 nm, Scan speed: 12,000 nm min^−1^, EX Slit: 5 nm, EM Slit: 5 nm, PMT Voltage: 700 V, Contour interval: 10 nm, Temperature: room temperature.

### Preparation of probe TPB

TPB was synthesized by the scheme showed in Figure S1 based on a previously reported method^20^, which was confirmed by FT-IR, UV–Vis, ^1^H NMR and ESI-MS. FT-IR: characteristic absorption peaks at 3432 cm^−1^ (C–H of aryl), 1699 cm^−1^ (C=O of carboxyl), 1597 cm^−1^ (N=N), 1499 cm^−1^ (aryl), 1253 cm^−1^ (aryl-N). UV–Vis: λ_max_ 355 nm (azobenzene). ^1^H NMR (600 MHz, CDCl_3_) δ (ppm): 8.71 (s, 3 H), 8.65–8.68 (s, 6 H), 7.94–7.95 (d, 6 H), 7.25 (s, 3 H), 7.0–7.1 (d, 6 H), 5.22 (s, 6 H). HRMS (*m*/*z*, ESI): [M]^−^ calcd. Found 971.2133 (TPB), 703.1660 (TPB-C_14_N_2_O_4_H_8_), 485.1024 (TPB-C_22_N_4_O_9_H_22_). All supplementary data used for confirmation can be found in Figures S2–S5 and Table S1.

### General procedure for fluorescent spectra measurement

Tetrahydrofuran, as a solvent, was used to prepare the probe TPB solution (0.06 mmol L^−1^), abamectin B_1_ solution (1.00 mg L^−1^), and DDH solution (0.06 mmol L^−1^). 0.5832 g TPB was added to a volumetric flask and mixed with tetrahydrofuran solution to 100 mL to get probe TPB solution (6.00 mmol L^−1^). Then, 1 mL TPB (6.00 mmol L^−1^) was added to a volumetric flask and mixed with tetrahydrofuran solution to 100 mL to get probe TPB solution (0.06 mmol L^−1^). 0.1716 g DDH was added to a volumetric flask and mixed with tetrahydrofuran solution to 100 mL to get probe DDH solution (6.00 mmol L^−1^). Then, 1 mL DDH (6.00 mmol L^−1^) was added to a volumetric flask and mixed with tetrahydrofuran solution to 100 mL to get DDH solution (0.06 mmol L^−1^). 1.0 mg abamectin B_1_ was added to a volumetric flask and mixed with tetrahydrofuran solution to 1 L to get abamectin B_1_ solution (1.0 mg L^−1^).

The appropriate excitation wavelengths and emission wavelengths of TPB in the absence and existence abamectin B_1_ and DDH were analyzed by fluorescent spectra respectively. One 10 mL colorimetric tube was filled with probe TPB (1.0 mL, 0.06 mmol L^−1^) and avermectin B_1_ (0.2 mL, 1.0 mg L^−1^). The other was only filled with probe TPB (1.0 mL, 0.06 mmol L^−1^). One 10 mL was only filled with DDH (1.0 mL, 0.06 mmol L^−1^). Then, they all fixed with tetrahydrofuran to 10 mL. For each sample in colorimetric tubes, test condition of the fluorescence intensity was as follows: sample mixed time: 10 s, Sampling interval: 5 nm, Scan speed: 12,000 nm min^−1^, EX Slit: 5 nm, EM Slit: 5 nm, PMT Voltage: 700 V, Contour interval: 10 nm, Temperature: room temperature, EX WL: 200–600 nm, EM WL: 200–600 nm.

In order to explore the quantitative relationship between TPB and avermectin. The fluorescence intensity of the reacted product at 420 nm were examined at different concentrations of abamectin B_1_ (0.00, 0.02, 0.04, 0.06, 0.08, 0.10, 0.12 mg L^−1^). Test condition of the fluorescence intensity was same as that of probe TPB, except EX WL: 360 nm and EM WL: 200–700 nm.

### Establishment and validation of the analysis method

To obtain the ideal pH condition of test, considering the emergence of esterification at pH below 5.0 and salt forming at pH above 5.0 for avermectin B_1_, the effect of pH 5.0–9.0 on the analysis result was investigated. Five 10 mL colorimetric tubes were filled with probe TPB (1.0 mL, 0.06 mmol L^−1^) and avermectin B_1_ (0.2 mL, 1.0 mg L^−1^), and fixed to 10 mL with pH 5.0–9.0 phosphate buffer (0.2 M) at room temperature, respectively. For each sample in colorimetric tubes, test condition of the fluorescence intensity was same as that of probe TPB, except EX WL: 360 nm and EM WL: 420 nm. pH 6.0 phosphate buffer (0.2 M) was found as an ideal pH condition and used in following tests.

To obtain the ideal mount of phosphate buffer, the effect of 0.2–1.2 mL phosphate buffer (0.2 M, pH 6.0) was investigated. Six 10 mL colorimetric tubes were filled with probe TPB (1.0 mL, 0.06 mmol L^−1^) and avermectin B_1_ (0.2 mL, 1.0 mg L^−1^), and fixed with 0.2, 0.4, 0.6, 0.8, 1.0 and 1.2 mL phosphate buffer (0.2 M, pH 6.0) at room temperature, respectively. For each sample in colorimetric tubes, test condition of the fluorescence intensity was same as that of probe TPB, except EX WL: 360 nm and EM WL: 420 nm. 0.8 mL phosphate buffer (0.2 M, pH 6.0) was found as an ideal mount and further applied in followed tests.

To establish the relationship between probe TPB and abamectin B_1_, at room temperature, 0.0 mL, 0.1, 0.2, 0.3, 0.4, 0.5 and 0.6 mL abamectin B_1_ (1.0 mg L^−1^) were added to probe TPB (1.0 mL, 0.06 mmol L^−1^), mixed with 0.8 mL phosphate buffer (0.2 M, pH 6.0) and fixed with tetrahydrofuran to 10 mL, respectively. For each sample, test condition of the fluorescence intensity was same as that of part 2.4, except EX WL: 360 nm, EM WL: 200–700 nm.

To validate the method under analytical control, the limit of detection (LOD), limit of quantification (LOQ), precision and linear range were implemented according to the previous methods^21,22^. A 10 mL colorimetric tubes was mixed with abamectin B_1_ (0.20 mL, 1.0 mg L^−1^) and probe TPB (1.0 mL, 0.06 mmol L^−1^), mixed with 0.8 mL phosphate buffer (0.2 M, pH 6.0), fixed with tetrahydrofuran to 10 mL and fluorescently detected according to that of probe TPB, except EX WL: 360 nm and EM WL: 420 nm. Eleven groups of parallel experiments were conducted. LOD and LOQ were calculated using the formulas shown in Eqs. (1) – (2). Precision was evaluated using relative standard deviation (RSD). The linear range was from LOQ to the maximum measured value.

1 $$ {\text{LOD}} = 3\delta /k $$

*δ*: standard deviation of the experiments, *k*: slope for the range of the linearity.

2 $$ {\text{LOQ}} = 10 \times \delta $$

*δ*: standard deviation of the experiments.

To perform the recovery rate, the standard addition method was chosen for detection of abamectin B_1_ in apple samples. Abamectin B_1_, at concentrations of 4.4 μg L^−1^, 8.8 μg L^−1^, 44 μg L^−1^, were added to the apple samples and fluorescence responses were introduced to detect these pesticides according to that of probe TPB, except EX WL: 360 nm and EM WL: 420 nm. Level of abamectin B_1_ was calculated according to the linear regression equation of this work. And the recovery rate was calculated using the formulas shown in Eq. (3).

3 $$ {\text{Recovery}}\;{\text{rate}}\;(\% ) = 100 \times (C_{A} - C_{B} )/C_{S} $$

*C*_*A*_: abamectin B_1_ value of apple samples filled with the standard abamectin B_1_, *C*_*B*_: abamectin B_1_ value of apple samples filled without the standard abamectin B_1_, *C*_*S*_: the filled standard abamectin B_1_ value.

### Preparation and analysis of apple samples

*Malus pumila* mill, Qinguan and Huangxiangjiao were purchased from local Xingfu Supermarket (Beijing, China) and analyzed. Apple samples were prepared according to a published method^23^. Each sample (20.0 g) was respectively added with acetonitrile (10.0 mL) and vigorously shaken for 2.0 min by a vortex mixer. Then, each mixture was mixed with 4.0 g anhydrous magnesium sulfate anhydrous and 1.0 g sodium chloride and shook for another 1.0 min. Following centrifugation at 4000 rpm for 5.0 min, 2.0 mL of the upper layer was transferred to a 20 mL volumetric flask and filled with tetrahydrofuran to obtain tested *Malus pumila* mill, Qinguan and Huangxiangjiao samples. These samples were fluorescently detected according to that of probe TPB, except EX WL: 360 nm and EM WL: 420 nm, respectively. Value of abamectin B_1_ was calculated according to the linear regression equation of this work.

## Supplementary Information

Supplementary Information.

## References

[1] Rúbies A, Antkowiak S, Granados M, Companyó R, Centrich F (2015). Determination of avermectins: A QuEChERS approach to the analysis of food samples. Food Chem..

[2] Egerton JR (1980). 22, 23-dihydroavermectin B_1_, a new broad-spectrum antiparasitic agent. Br. Vet. J..

[3] Shen H (2017). Determination and correlation of Avermectin B_1a_ solubility in different binary solvent mixtures at temperatures from (283.15 to 313.15) K. J. Chem. Thermodyn..

[4] Teixeira RA, Flores DHÂ, da Silva RCS, Dutra FVA, Borges KB (2018). Pipette-tip solid-phase extraction using poly(1-vinylimidazole-co-trimethylolpropane trimethacrylate) as a new molecularly imprinted polymer in the determination of avermectins and milbemycins in fruit juice and water samples. Food Chem..

[5] Zhou QZ (2019). The effects and mechanism of using ultrasonic dishwasher to remove five pesticides from rape and grape. Food Chem..

[6] Zhan J (2013). Multi-class method for determination of veterinary drug residues and other contaminants in infant formula by ultra performance liquid chromatography-tandem mass spectrometry. Food Chem..

[7] Lemos MAT (2016). Development, validation, and application of a method for selected avermectin determination in rural waters using high performance liquid chromatography and fluorescence detection. Ecotoxicol. Environ. Safe.

[8] Zhao WD, Zheng WJ, He Y, Wan YP, Wang SL (2009). Determination of avermectin residues in animal products by ELISA. Food Res. Dev..

[9] Park JH, Abd El-Aty AM, Rahman MM, Choi JH, Shim JH (2013). Application of hollow-fiber-assisted liquid-phase microextraction to identify avermectins in stream water using MS/MS. J. Sep. Sci..

[10] Ni TT (2019). Development of a broad-spectrum monoclonal antibody-based indirect competitive enzyme-linked immunosorbent assay for the multi-residue detection of avermectins in edible animal tissues and milk. Food Chem..

[11] De Souza Santos Cheibub AM, Silva Bahiense de Lyra E, Pereira Netto AD (2019). Development and validation of a method for simultaneous determination of trace levels of five macrocyclic lactones in cheese by HPLC-fluorescence after solid–liquid extraction with low temperature partitioning. Food Chem..

[12] Zhao M (2020). Far-red to near-infrared fluorescent probes based on silicon-substituted xanthene dyes for sensing and imaging. Trend Anal. Chem..

[13] Wu D (2019). Comparative analysis of the interaction of mono-, dis-, and tris-azo food dyes with egg white lysozyme: A combined spectroscopic and computational simulation approach. Food Chem..

[14] An YL, Tan HR, Zhao SY (2017). Silver carbonate mediated oxidative dehydrogenation of aromatic amines to produce aromatic azo compounds. Chin. J. Org. Chem..

[15] Wu XL, Wang PS, Hou SY, Wu PL, Xue J (2019). Fluorescence sensor for facile and visual detection of organophosphorus pesticides using AIE fluorogens–SiO_2_–MnO_2_ sandwich nanocomposites. Talanta.

[16] Hussein BHM, Khairy GM, Kamel RM (2016). Fluorescence sensing of phosdrin pesticide by the luminescent Eu(III)- and Tb(III)- bis (coumarin-3-carboxylic acid) probes. Spectrochim. Acta A..

[17] Kestwal RM, Bagal-Kestwal D, Chiang BH (2015). Fenugreek hydrogel–agarose composite entrapped gold nanoparticles for acetylcholinesterase based biosensor for carbamates detection. Anal. Chim. Acta.

[18] Wang JJ (2018). A novel reaction-based fluorescent probe for the detection of cysteine in milk and water samples. Food Chem..

[19] Wang Z, Beier RC, Shen J (2017). Immunoassays for the detection of macrocyclic lactones in food matrices—A review. Trend Anal. Chem..

[20] Eubank JF (2012). On demand: The singular rht net, an ideal blueprint for the construction of a Metal-Organic Framework (MOF) platform. Angew. Chem. Int. Ed..

[21] Yan HM (2019). A water-soluble fluorescent probe for the detection of thiophenols in water samples and in cells imaging. Spectrochim. Acta A.

[22] Zenaida G, Olga MP, Álvaro G, José DC, Belén A (2012). Quantitative determination of wine polysaccharides by gas chromatography–mass spectrometry (GC–MS) and size exclusion chromatography (SEC). Food Chem..

[23] Wilkowska A, Biziuk M (2011). Determination of pesticide residues in food matrices using the QuEChERS methodology. Food Chem..

[24] Yuan L, Lin W, Zheng K, He L, Huang W (2013). Far-red to near infrared analyte-responsive fluorescent probes based on organic fluorophore platforms for fluorescence imaging. Chem. Soc. Rev..

[25] Ren TB (2018). A general method to increase Stokes shift by introducing alternating vibronic structures. J. Am. Chem. Soc..

[26] Wang ZL (2020). Two ultrafast responsive isolongifolanone based fluorescent probes for reversible and sensitive visualization of toxic BF_3_ in solution and in gas phase. Sensor Actuator B Chem..

