# Supplementary material for: A highly sensitive octopus-like azobenzene fluorescent probe for determination of abamectin B1 in apples
Source: Sci Rep. 2021 Feb 25;11:4655. doi: 10.1038/s41598-021-84221-w (PMC7907368; doi:10.1038/s41598-021-84221-w)
Supplement: Supplementary file 1 — Supplementary Information. [file 41598_2021_84221_MOESM1_ESM.doc]

**A highly sensitive octopus-like azobenzene fluorescent probe for determination of abamectin B_1_ in apples**

Zhenlong Guo^1,2,3^, YiFei Su^2^, Kexin Li^2^, MengYi Tang^2^, Qiang Li^1, 3*^, Shandong Xu^1*^

^1^*Department of Chemistry*, *College of Science, Beijing Forestry University, Beijing 100083, China*

^2^*College of Biological Sciences and Biotechnology, Beijing Forestry University, Beijing 100083, China*

^3^*Beijing Key Laboratory of Forest Food Processing and Safety, Beijing 100083, China*

^*^Corresponding author:

*E-mail address*: liqiang@bjfu.edu.cn (Qiang Li), Phone: +86-137-18679671;

*E-mail address*: [xushd@](mailto:susansun@)bjfu.edu.cn (Shandong Xu), Phone: +86-136-61232609.

**Figure. S1**. Synthesis scheme of TPB


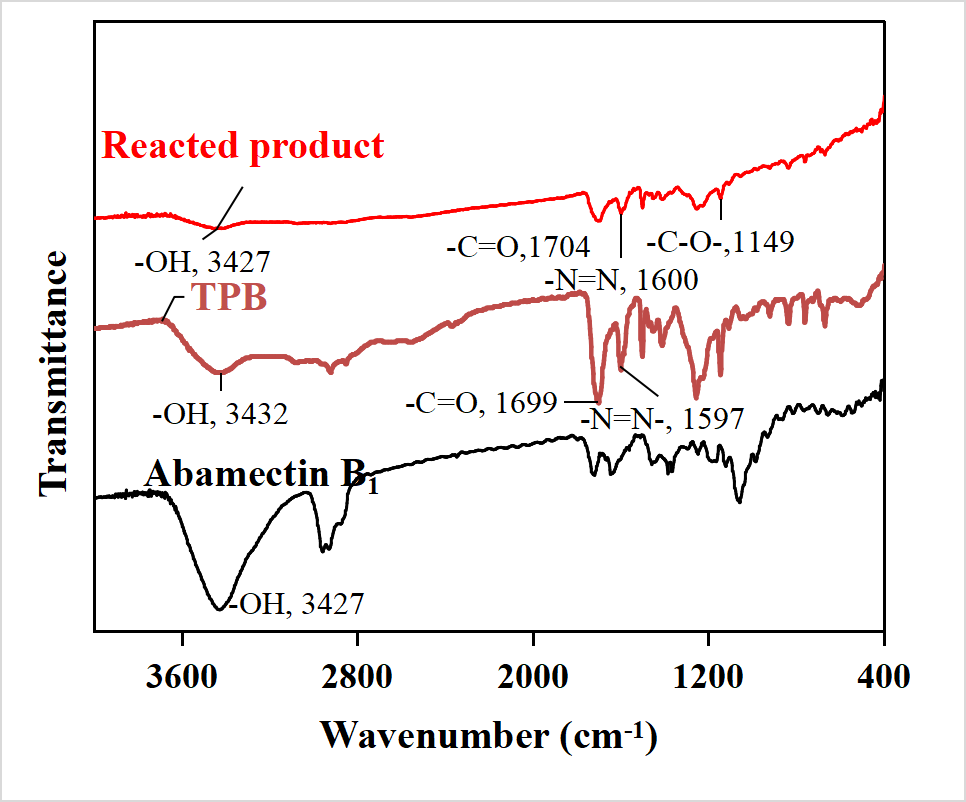


**Figure. S2**. FT-IR spectra of TPB, abamectin B_1_ and the reacted product


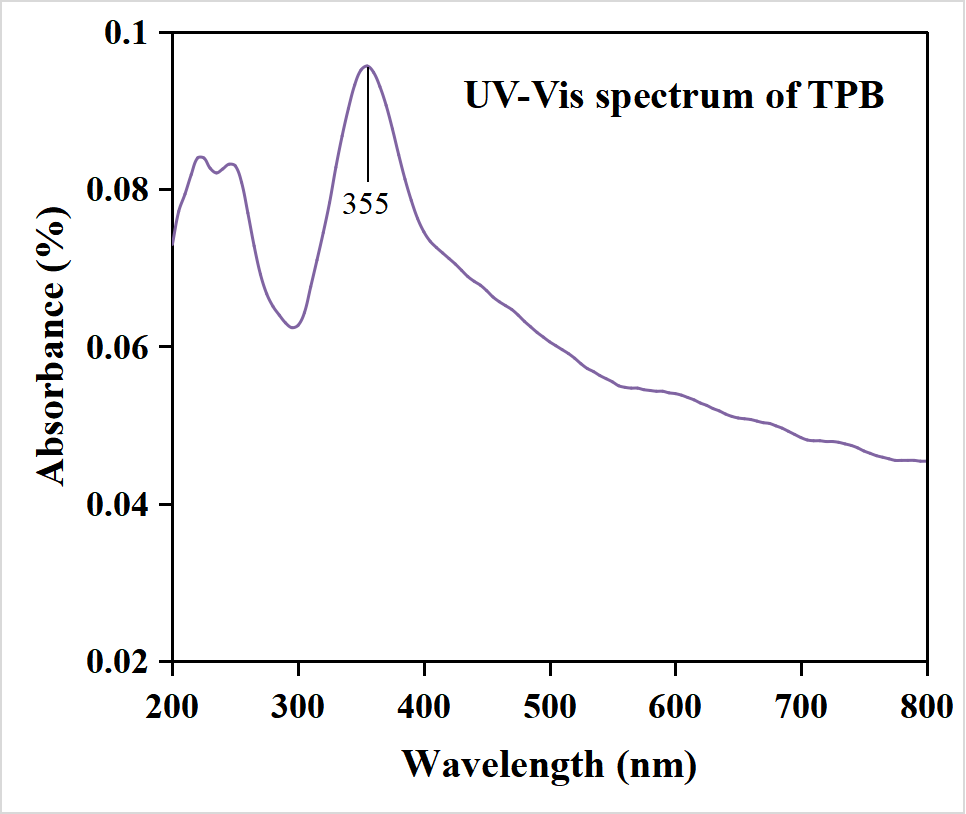


**Figure. S3**. UV-Vis spectra of TPB

**
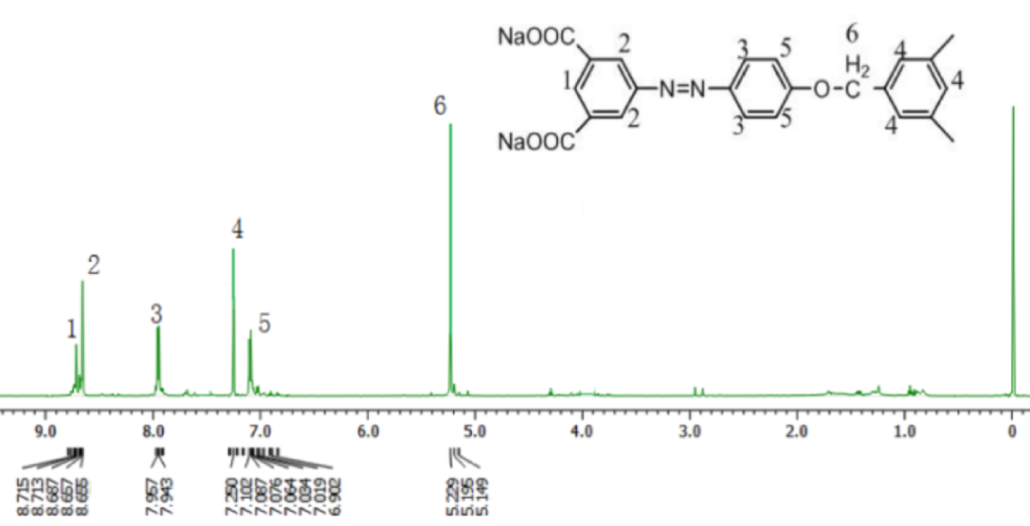
**

**Figure. S4**. ^1^H NMR spectra of TPB

**Figure. S5**. ESI-MS spectra of TPB

**Figure. S6**. ESI-MS spectra of the reacted product


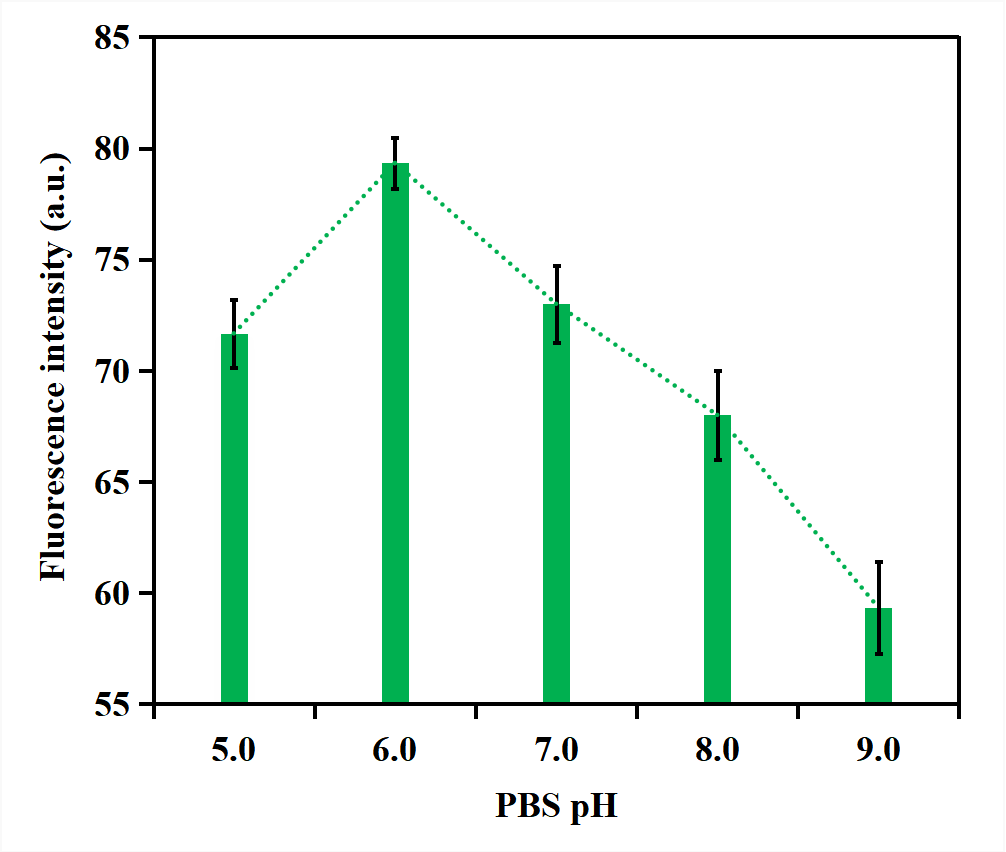


**Figure. S7**. Fluorescent intensity of the reacted product at pH 5.0-9.0


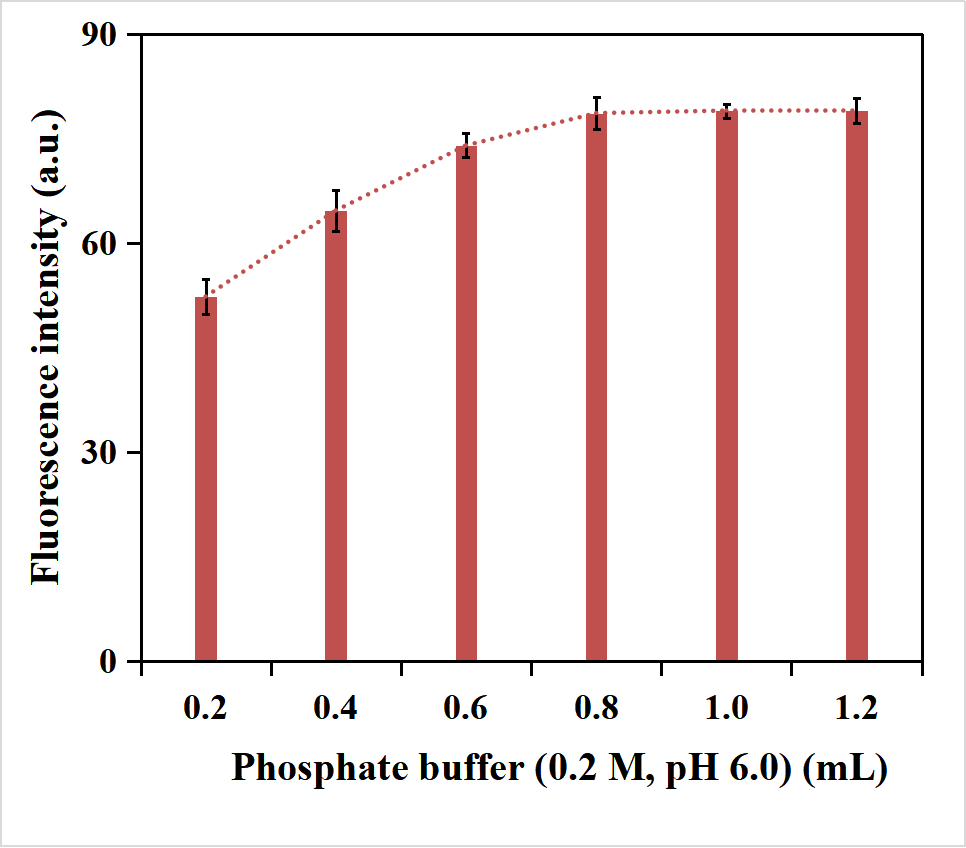


**Figure. S8**. Fluorescent intensity of the reacted product at 0.2-1.2 mL phosphate buffer (0.2 M, pH 6.0)

**Figure. S9**. Structure of abamectin B_1_

**Table S1**. Number and position of hydrogen atoms based on integral values and spectra of ^1^H NMR of TPB

| Shift value (ppm) | Peak types | Number of hydrogen atoms | Position on the structure of TPB |
| --- | --- | --- | --- |
| 8.71 | s | 3 H | 1 |
| 8.65-8.68 | s | 6 H | 2 |
| 7.94-7.95 | d | 6 H | 3 |
| 7.25 | s | 3 H | 4 |
| 7.0-7.1 | d | 6 H | 5 |
| 5.22 | s | 6 H | 6 |

**Table S2**. Fluorescent detected results of eleven parallel experiments

| Order | 1 | 2 | 3 | 4 | 5 | 6 | 7 | 8 | 9 | 10 | 11 |
| --- | --- | --- | --- | --- | --- | --- | --- | --- | --- | --- | --- |
| Y (a.u.) | 79.91 | 79.90 | 79.92 | 79.89 | 79.88 | 79.92 | 79.92 | 79.90 | 79.88 | 79.92 | 79.91 |
| SD (%) | 1.8 | | | | | | | | | | |
| RSD (%) | 2.2 | | | | | | | | | | |
| LOD (μg L^-1^) | 4.4 | | | | | | | | | | |
| LOQ (μg L^-1^) | 1.3 | | | | | | | | | | |

**Table S3**. Comparison was conducted between our fluorescent probe method and those described previously.

| **Analysis technique** | **Recovery (%)** | **Linear Range (μg L^-1^)** | **LOQ (μg L^-1^)** | **Ref.** |
| --- | --- | --- | --- | --- |
| HPLC-UV | 88.0-110.0 | 25.0-750.0 | 25.0 | 4 |
| HPLC-MS | 106.0-135.0 | - | 200.0 | 6 |
| HPLC-FLD | 93.0-119.0 | - | 0.2 | 7 |
| ELISA | 62.3-94.3 | - | 1.0 | 8 |
| LC-MS/MS | 80.0-94.0 | 0.2-100.0 | 0.5 | 9 |
| Fluorescent probe | 98.2-104.5 | 4.4-60.0 | 4.4 | This work |
